# 2406 The microbial-derived short-chain fatty acid butyrate directly and differentially inhibits gut T helper cell subset activation and proliferation

**DOI:** 10.1017/cts.2018.134

**Published:** 2018-11-21

**Authors:** Jon Kibbie, Stephanie Dillon, Moriah Castleman, Jay Liu, Martin McCarter, Cara Wilson

**Affiliations:** University of Colorado at Denver

## Abstract

OBJECTIVES/SPECIFIC AIMS: A hallmark of progressive HIV-1 infection is the massive activation and depletion of the gut barrier protective CD4 T helper subsets (Th17 and Th22) in the intestinal mucosa. The loss of these cells is thought to contribute to microbial translocation and systemic immune activation that occurs during chronic infection. In addition to the loss of protective Th subsets, we previously showed that chronically HIV-1 infected individuals have an altered colonic mucosal microbiome, which is in part characterized by a lower relative abundance of bacteria that produce the short-chain fatty acid butyrate in conjunction with increased relative abundance of gram-negative pathobionts. This dysbiosis was linked to markers of mucosal and systemic immune activation in these individuals. Following up on these clinical observations, we sought to understand how a loss of butyrate might contribute to HIV-associated inflammation. Initial studies showed that the addition of butyrate to cultured lamina propria mononuclear cells (LPMC) resulted in decreased pathobiont-driven gut T cell activation, HIV-1 infection levels and production of IL-17 and IFNy. Since the gut barrier protective Th17 and Th22 subsets are preferentially infected and depleted, which is critical to HIV-1 pathogenesis, we wanted to determine the mechanism by which butyrate modulates activation of these important Th subsets in the gut. METHODS/STUDY POPULATION: Total LPMCs or purified LP CD4 T cells were isolated from human jejunal tissue (n=3–6), labeled with CFSE and cultured with TCR/CD28 beads to mimic APC driven T cell activation, with the addition of butyrate at physiologic doses(0–2 mM). Four days after culture, secreted cytokine(IL-17 and IFNy) levels were measured by ELISA. Cells were then short-term (4 hr) mitogenically stimulated (PMA/Ionomycin) in the presence of a golgi transport inhibitor. Total CD4 T cell activation (CD38+/HLA-DR+, CD25+), proliferation (CFSElow), and frequencies of intracellular cytokines were measured by multi-color flow cytometry. Paired *t*-tests were performed to determine statistical significance. RESULTS/ANTICIPATED RESULTS: Butyrate inhibited LP CD4 T cell activation (*p*=0.013) and proliferation (*p*=0.015) within total LPMCs stimulated with TCR/CD28 beads in a dose-dependent manner, with significant activity starting at 0.125 mM. Quantification of total secreted cytokines revealed that butyrate significantly decreased both IL-17 and IFNy production after 4 days of culture at 0.0625 mM and 0.25 mM of butyrate, respectively. Assays using purified LP CD4 T cells demonstrated that butyrate directly decreased LP CD4 T cell activation, proliferation and cytokine production in response to TCR/CD28 stimulation. Studies on specific T helper subsets revealed that butyrate inhibited proliferation of Th17 cells at lower concentrations (IC50:0.147 mM) compared with Th1 (IC50:0.229 mM) and Th22 (IC50:0.258 mM) and Th non-IL-22/IL-17/IFNy producing (IC50:2.14 mM) subsets. In addition, it appeared there was a paradoxical increase of HIV-1 infection levels at lower concentrations of butyrate (0.125 mM). DISCUSSION/SIGNIFICANCE OF IMPACT: The addition of butyrate to activated LP CD4 T cells decreases TCR-mediated activation in a dose-dependent manner, and butyrate acts directly on purified LP CD4 T cell populations independent of other cell populations. Butyrate differentially inhibited the proliferation of Th17, Th1, and Th22 subsets, with Th17 cells being the most sensitive to butyrate but increased the infection levels of all T helper subsets at low concentrations. Further studies are needed to determine the mechanism of butyrate’s actions on LP Th cells and the sensitivity of Th17 cells to the inhibitory effects of butyrate. These results could help direct targeted manipulation of the colonic microbiome of HIV-1 infected individuals to help resolve inflammation and limit the impact of the infection in the gut mucosa and systemically.

